# Homologization of the Flight Musculature of Zygoptera (Insecta: Odonata) and Neoptera (Insecta)

**DOI:** 10.1371/journal.pone.0055787

**Published:** 2013-02-14

**Authors:** Sebastian Büsse, Cécile Genet, Thomas Hörnschemeyer

**Affiliations:** Department of Morphology, Systematic and Evolutionary Biology, J.F. Blumenbach Institute for Zoology and Anthropology, Georg-August-University Göttingen, Göttingen, Germany; Institut de Biologia Evolutiva - Universitat Pompeu Fabra, Spain

## Abstract

Among the winged insects (Pterygota) the Dragonflies and Damselflies (Odonata) are unique for several reasons. Behaviourally they are aerial predators that hunt and catch their prey in flight, only. Morphologically the flight apparatus of Odonata is significantly different from what is found in the remaining Pterygota. However, to understand the phylogenetic relationships of winged insects and the origin and evolution of insect flight in general, it is essential to know how the elements of the odonatan flight apparatus relate to those of the other Pterygota. Here we present a comprehensive, comparative morphological investigation of the thoracic flight musculature of damselflies (Zygoptera). Based on our new data we propose a homologization scheme for the thoracic musculature throughout Pterygota. The new homology hypotheses will allow for future comparative work and especially for phylogenetic analyses using characters of the thoracic musculature throughout all winged insects. This will contribute to understand the early evolution of pterygote insects and their basal phylogenetic relationship.

## Introduction

Within the insects the Odonata arguably are the group with the most impressive flight skills (e.g. [1]). Each wing pair can be controlled independently and some species are even able to fly backwards [2]. Through these flight skills Odonata are the avian key predators among insects [1].

The unique flight abilities are also reflected in a unique morphology. The meso- and metathorax forms a functional unit, the ptero- or synthorax, which is tilted caudally by 45°. The pleurites are strongly enlarged in dorso-ventral direction, whereas, the tergites and sternites are unusually small if compared to other pterygotes [2]–[4].

The muscles responsible for the wing movement are connected via cap tendons and sclerites directly to the wings [5]. This exclusively direct mechanism of wing movement distinctly sets Odonata apart from all other winged insects; where the wing beat is done mainly through a system of indirect muscles, many of which are highly reduced or missing in the Odonata (e.g. [6]).

Several publications address the structures of the flight apparatus of Odonata [5], [7]–[10], the aerodynamics of odonatan flight [10]–[12], the mechanics [2] and function of the flight musculature and the mechanoreceptors of the wing [10] as well as the complexity of the wing venation [13]. All these publications deal mainly with representatives of Anisoptera. In total, the knowledge about the odonatan thorax morphology shows a distinct deficit for the Zygoptera, which we, therefore, focused our comparative investigation on.

Major research has been carried out by Asahina [7], who studied *Mnais strigata* Hagen, 1853 (Zygoptera), *Davidius nanus* (Sélys, 1869) (Anisoptera) and *Epiophlebia superstes* Sélys, 1889 (*Epiophlebia*). Ninomiya and Yoshizawa [14], investigated the skeletal morphology of *Coeliccia ryukyuensis ryukyuensis* Asahina, 1951 (Zygoptera), *Tanypteryx pryeri* (Sélys, 1889) (Anisoptera) and *Epiophlebia superstes*.

Presently there seems to be widespread agreement on ground pattern hypotheses for the wing base sclerites and for the flight musculature in Neoptera [15]–[18]. Even homologies between Ephemeroptera and Neoptera are mainly resolved [17], [19], while hypotheses on the homologies between Odonata and the remaining Pterygota are still under discussion [17], [19], [14], [10].

The aim of our comprehensive comparative investigation of the flight musculature of the Zygoptera is to identify variabilities among the Zygoptera and to establish homology hypotheses for the thoracic musculature of Odonata and Neoptera.

## Results

In the following descriptions of the musculature the condition in *Phyrrhosoma nymphula* (Fig. 1, 2, 3, 4, 5, 6, 7, 8) is used as a point of reference. This infomaration is supplemented with and compared to data from *Coenagrion puella*, *Enallagma cyathigerum*, *Ischnura elegans Calopteryx splendens* (Fig. 9, 10, 11, 12), *Platycnemis latipes*, *Platycnemis pennipes* and *Lestes viridis.*

**Figure 1:**
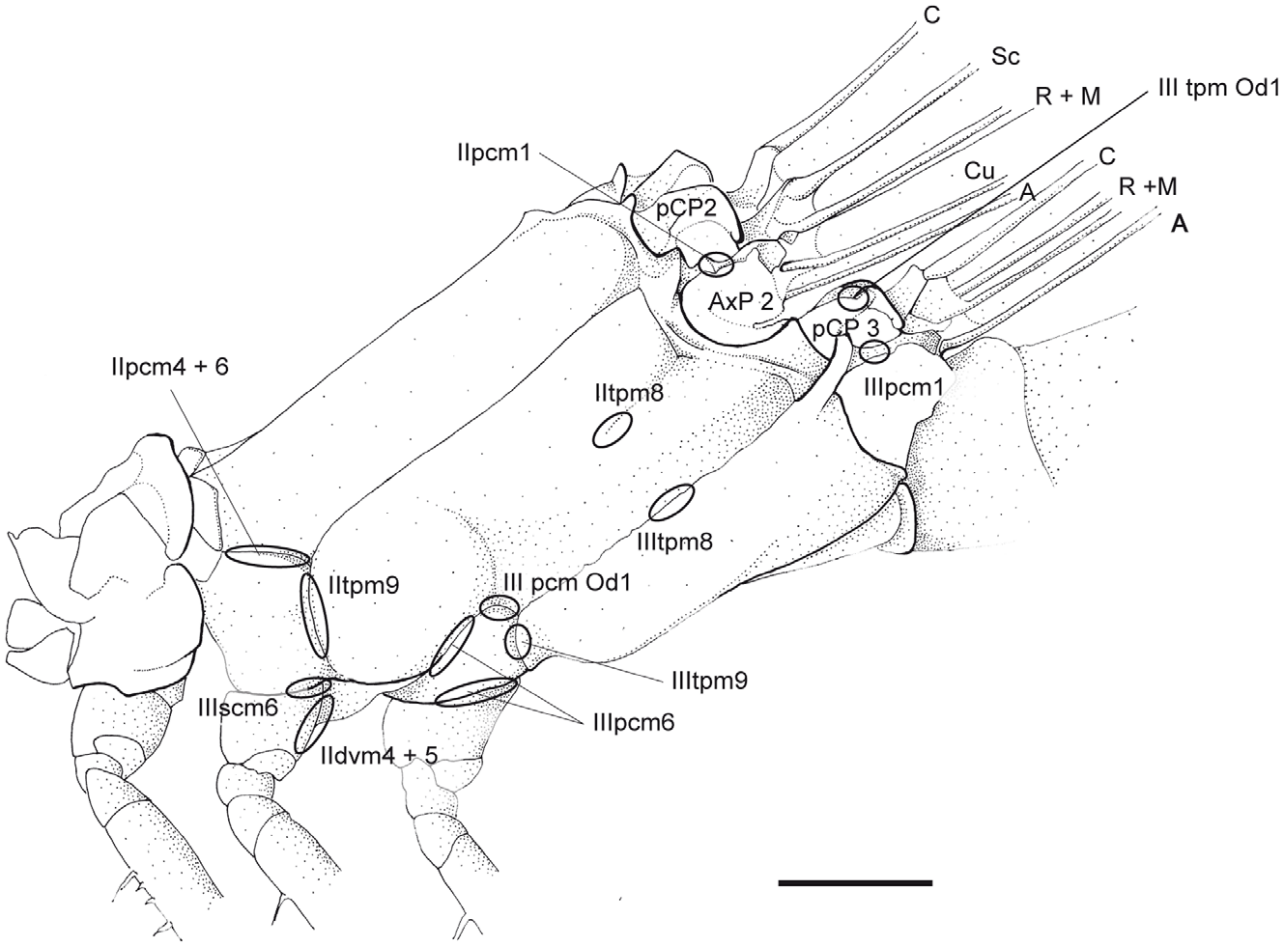
Thorax of *P. nymphula*, muscle attachment points as seen from outside, left, lateral view. Abbreviations: A – anal vein, AxP – axillary plate, C – costa, Cu – cubitus, M – media, pCP – proximale costal plate, R – radius, Sc – subcosta.

**Figure 2:**
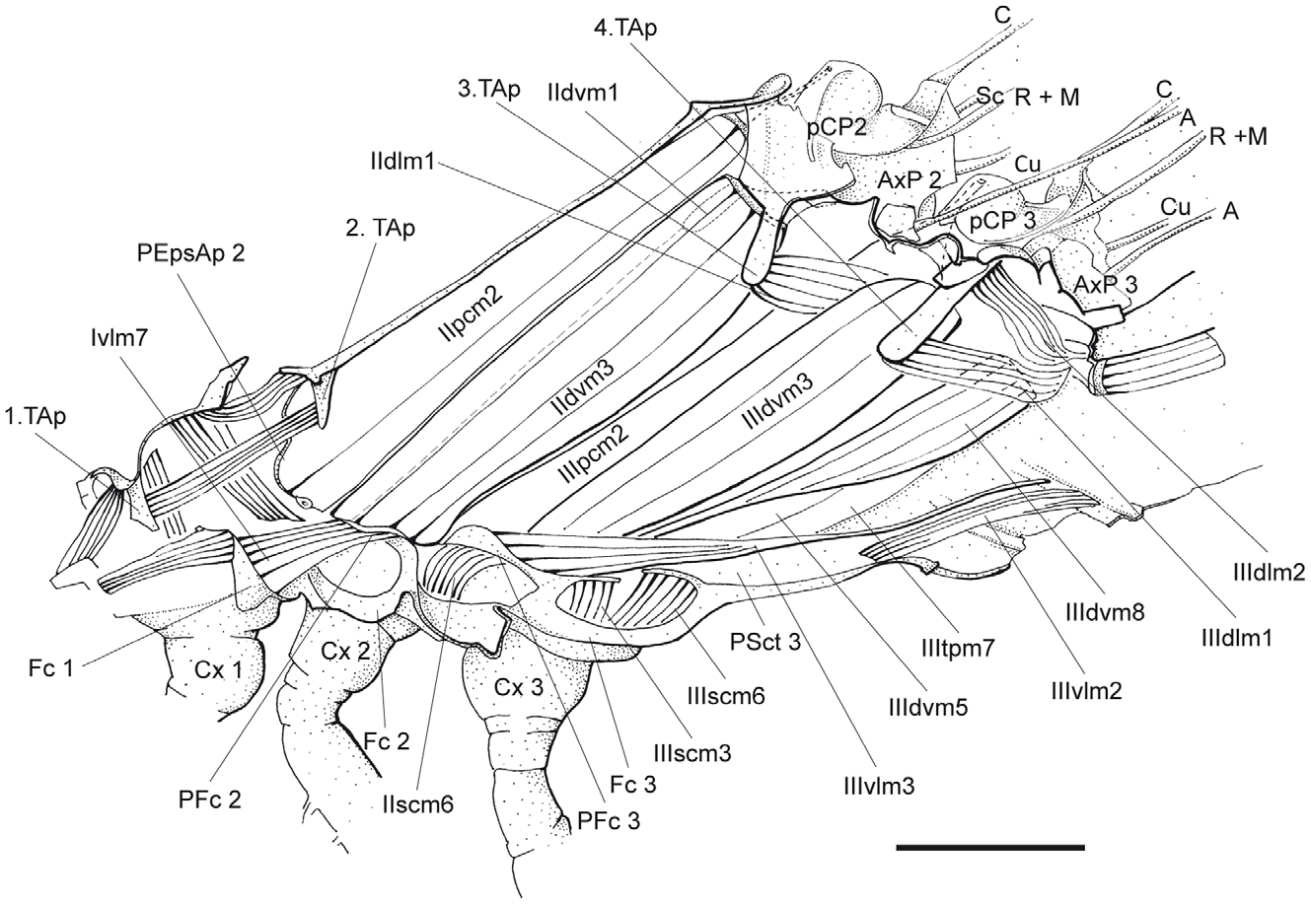
*P. nymphula*, innermost layer of thorax musculature, longitudinal cut, right. Abbreviations: A – anal vein, AxP – axillary plate, C – costa, Cu – cubitus, Cx – coxa, Fc – Furca, M – media, pCP – proximale costal plate, PEpsAp – preepisternal apodem, PFc – prefurca, PSct – prescutum, R – radius, Sc – subcosta, TAp – tergal aphophyse.

**Figure 3:**
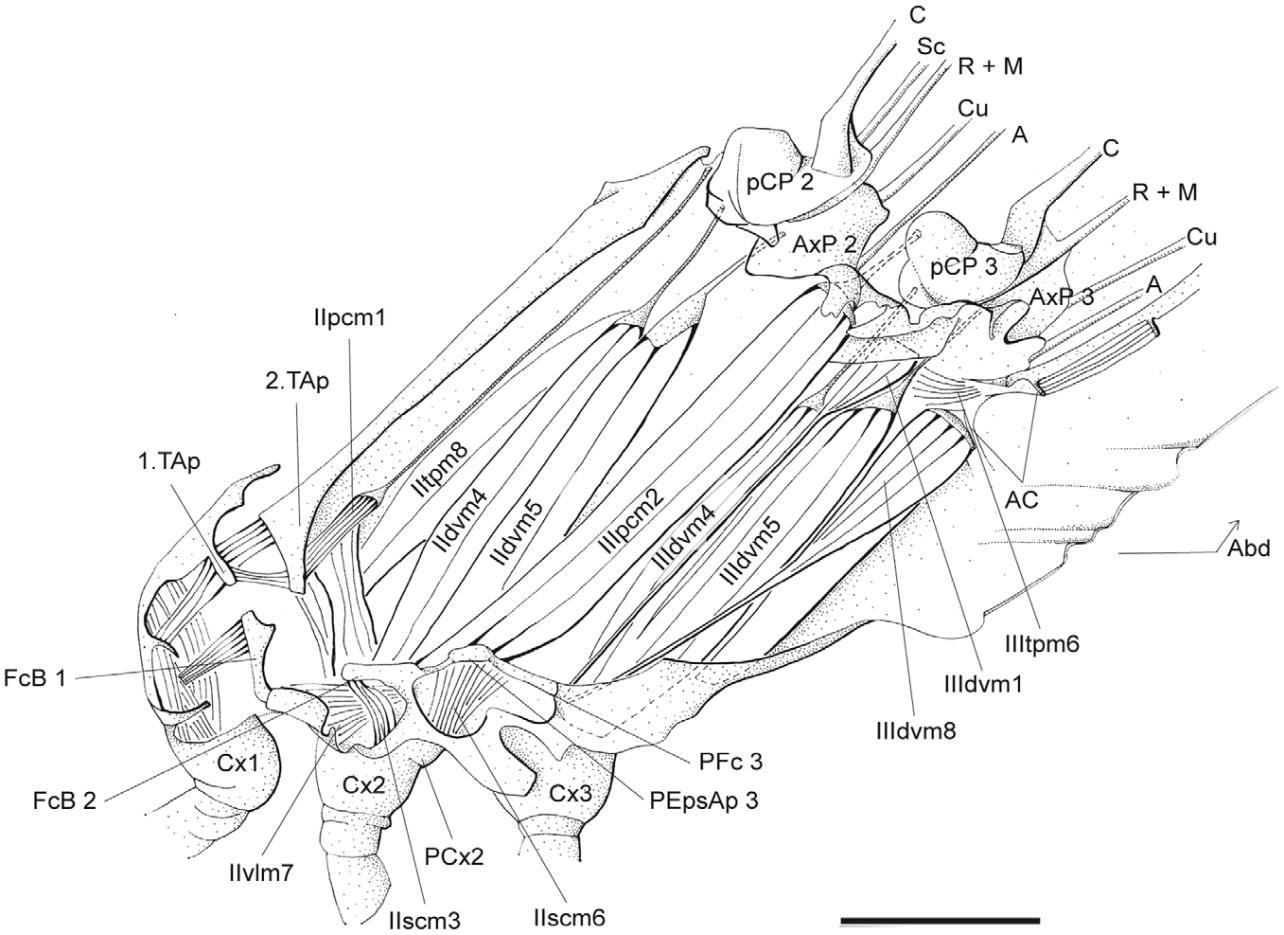
*P. nymphula*, thorax musculature, inner musculature removed, longitudinal cut, right. Abbreviations: A – anal vein, Abd – abdominal segment, AC – antecosta, AxP – axillary plate, C – costa, Cu – cubitus, Cx – coxa, FcB – furcalbranch, M – media, pCP – proximale costal plate, PCx – precoxa, PEpsAp – preepisternal apodem, PFc – prefurca, R – radius, Sc – subcosta, TAp – tergal aphophyse.

**Figure 4:**
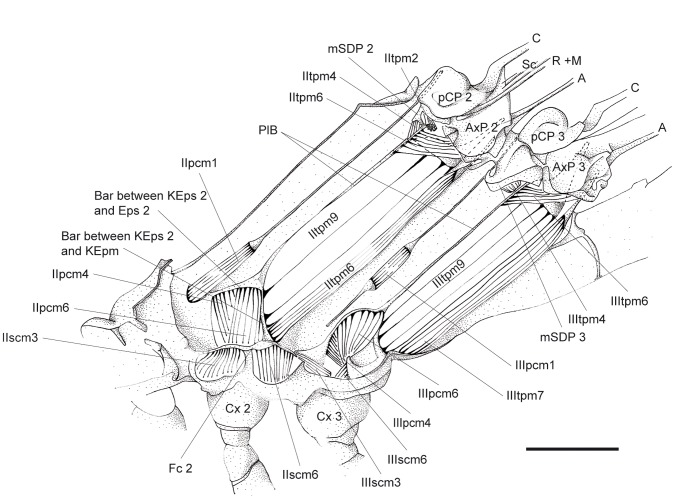
*P. nymphula*, lateral thorax musculature, longitudinal cut, right. Abbreviations: A – anal vein, AxP – axillary plate, C – costa, Cx – coxa, Eps – episternum, KEpm – katepimerom, KEps – katepisternum, M – media, mSDP - mediane semi-detached scutal plate, pCP – proximale costal plate, PlB - pleuralbar, R – radius, Sc – subcosta.

**Figure 5:**
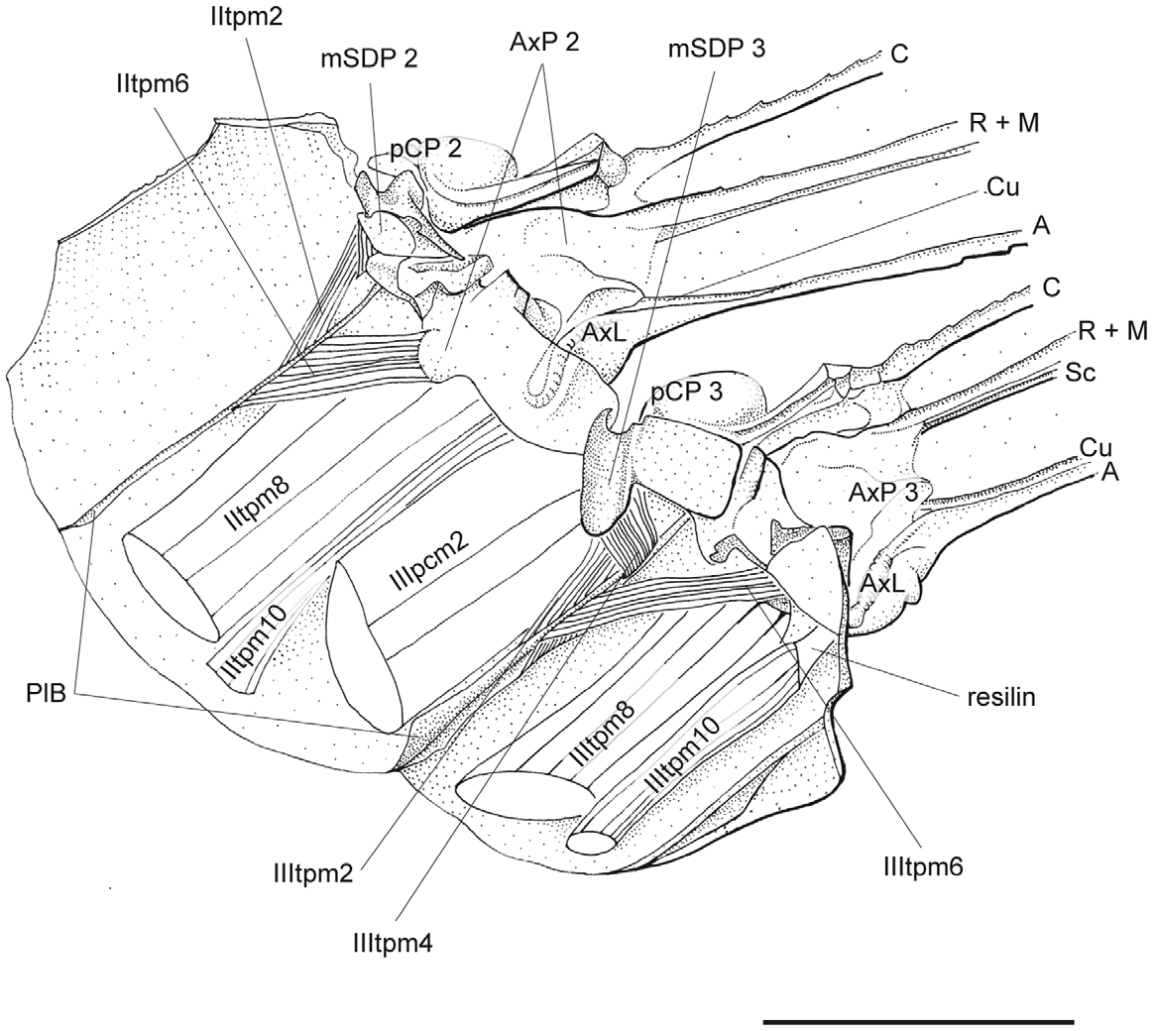
*P. nymphula*, detail of dorasl area of lateral flight musculature, longitudinal section, right. Abbreviations: A – anal vein, AxL – axillary ligament, AxP – axillary plate, C – costa, Cu – cubitus, M – media, mSDP - mediane semi-detached scutal plate, pCP – proximale costal plate, PlB - pleuralbar, R – radius, Sc – subcosta.

**Figure 6:**
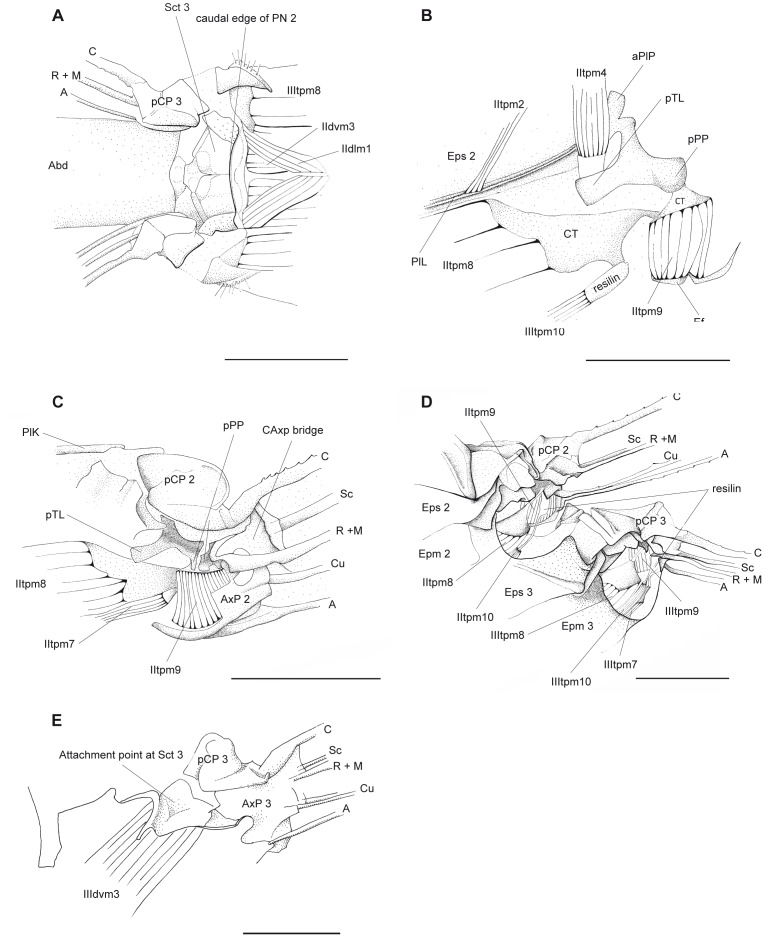
*P. nymphula*, thorax details. A Points of origin of muscles IIdlm1, IIIdvm3 and IIItpm9. Dorsal view. B Detail of lateral flight muscles, longitudinal section, right. C Attachment points of muscles IItpm9, IItpm6 and IItpm8. Longitudinal section, right. D Detail of wing articulation area of the meso- and metathorax, left lateral view. E Attachment of muscle III dvm3. Longitudinal section, right. Abbreviations: A – anal vein, aPlP - anterior pleural process, AxP – axillary plate, C – costa, CAxP bridge - costa-axillary plate bridge, CT – cap tendon, Cu – cubitus, Epm – epimeron, Eps – episternum, M – media, pCP - proximale costal plate, PIL - pleuralbar, PlK – pleuralkeel, PN – postnotum, pPP – posterior pleural process, pTL - posterior tergal levler, R – radius, Sc – subcosta, Sct – scutum.

**Figure 7:**
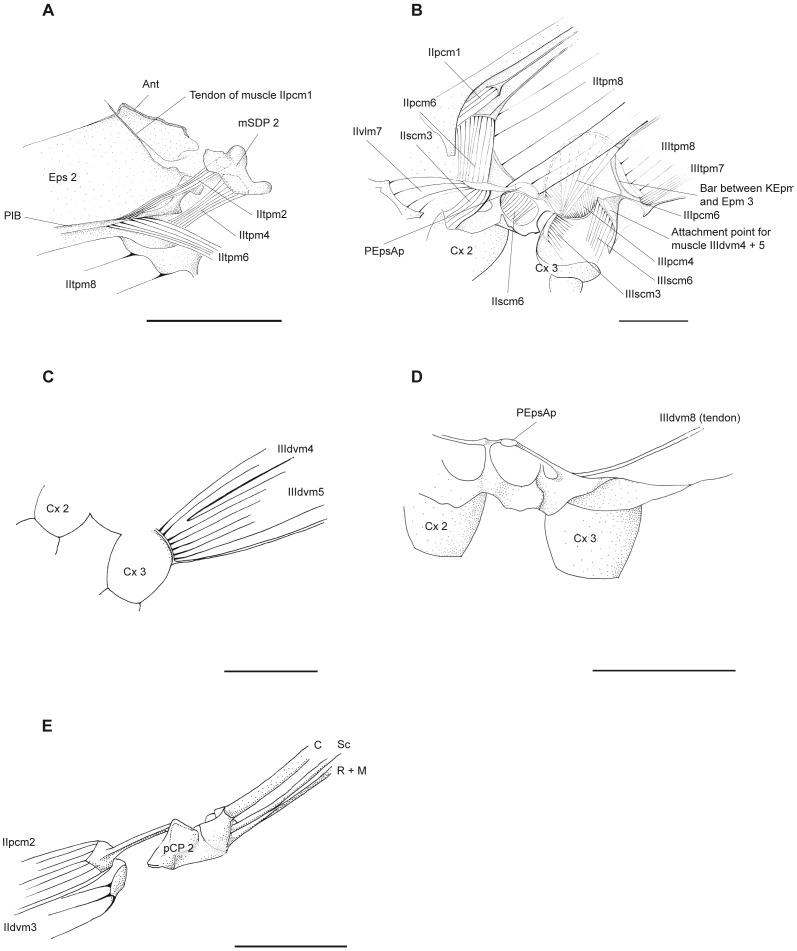
*P. nymphula*, details of muscle attachements. A–D Longitudinal section, right. A Attachments point of muscles IItpm4, IIItpm4, IIItpm6 and IItpm8. B Ventral thorax musculature. C Points of origin of muscles IIIdvm4 and IIdvm5. D Point of origin of muscle IIIdvm8. E Detail of attachment points of muscles IIpcm2, IIdvm3. Dorsal view, right. Abbreviations: Ant – antealar plate, C – costa, Cx – coxa, Epm – epimeron, Eps – episternum, KEpm – katepimerom, M – media, mSDP - mediane semi-detached scutal plate, PEpsAp – preepisternal apodem, pCP - proximale costal plate, PIB – pleuralbar, R – radius, Sc – subcosta.

**Figure 8:**
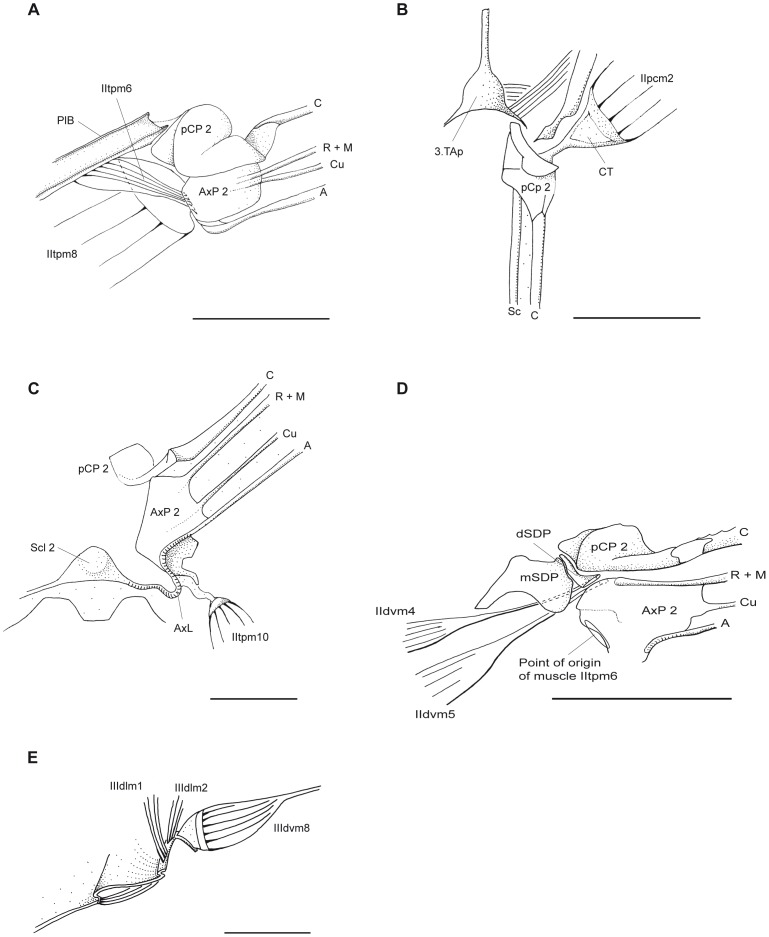
*P. nymphula*, details of muscle attachment points. A, D and E Longitudinal section, right. B and C Dorsal view, right. A Point of origin of muscle IItpm6. B Attachment point of muscle IIpcm2. C Attachment point of muscle IItpm10. D Points of origin of muscles IIdvm4, IIdvm5 and IItpm6. E Points of origin of muscles IIIdlm1, IIIdlm2 and IIIdvm8. Abbreviations: A – anal vein, AxL – axillary ligament, AxP – axillary plate, C – costa, CT – cap tendon, Cu – cubitus, dSDP - distal semi-detached scutal plate, M – media, mSDP - mediane semi-detached scutal plate, pCP – proximale costal plate, PlB – pleuralbar, R – radius, Sc – subcosta, Scl – scutellum, TAp – tergal aphophyse.

**Figure 9:**
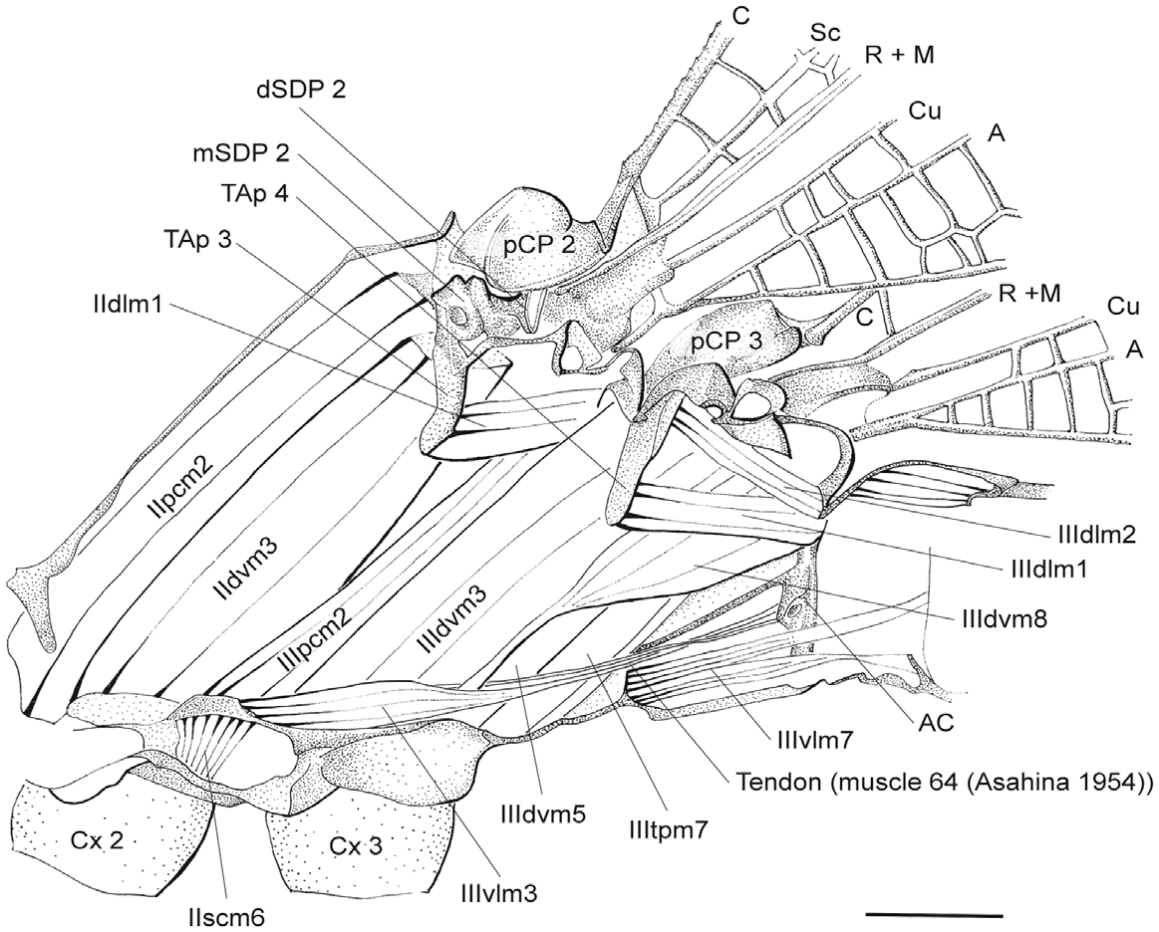
*C. splendens*, innermost layer of thorax musculature. Longitudinal cut, right. Abbreviations: A – anal vein, AC – antecosta, C – costa, Cu – cubitus, Cx – coxa, dSDP - distal semi-detached scutal plate, M – media, mSDP - mediane semi-detached scutal plate, pCP – proximale costal plate, R – radius, Sc – subcosta, TAp – tergal aphophyse.

**Figure 10:**
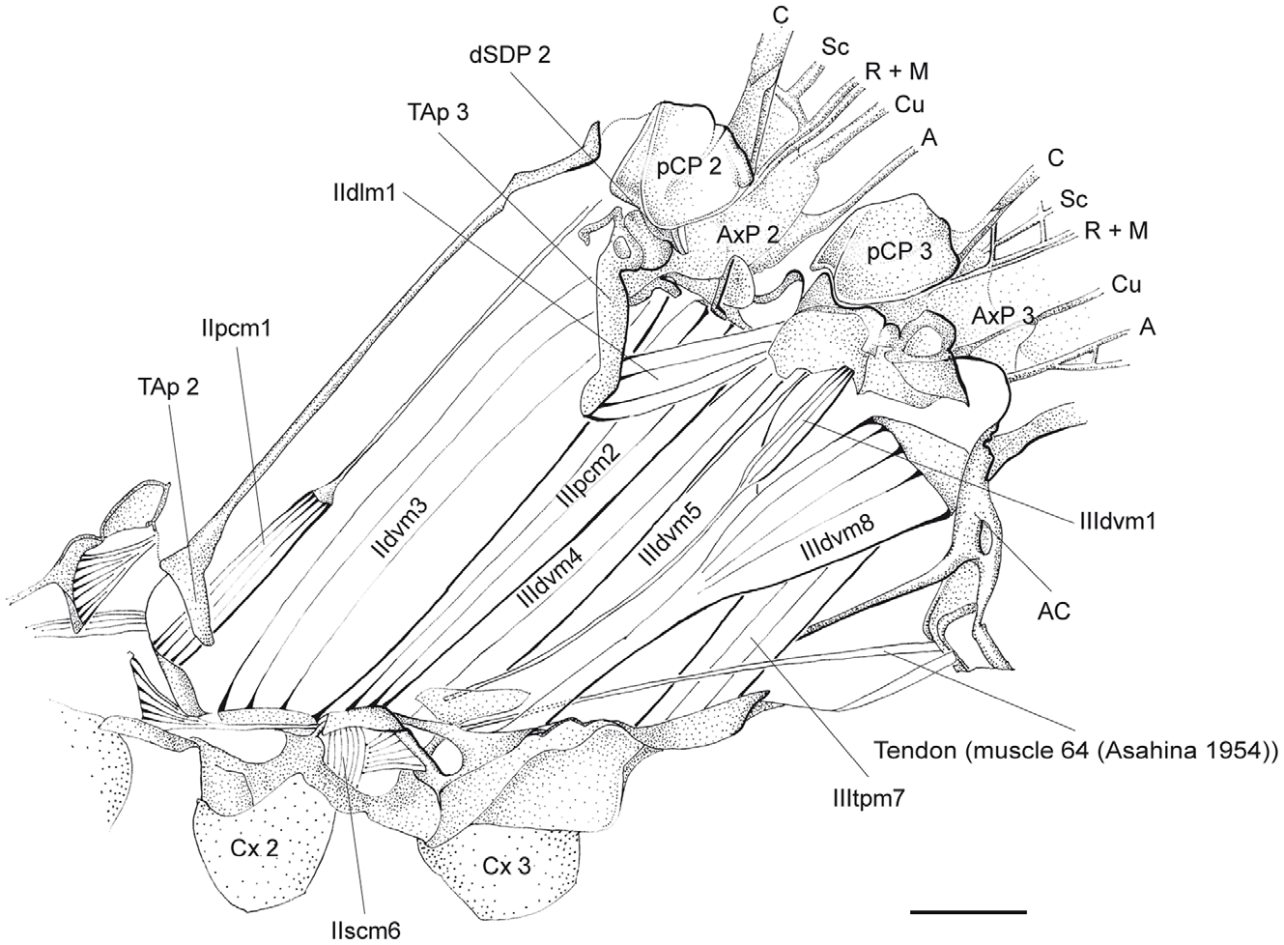
*C. splendens*, thorax musculature. Inner musculature removed, longitudinal cut, right. Abbreviations: A – anal vein, AC – antecosta, AxP – axillary plate, C – costa, Cu – cubitus, Cx – coxa, dSDP - distal semi-detached scutal plate, M – media, pCP – proximale costal plate, R – radius, Sc – subcosta, TAp – tergal aphophyse.

**Figure 11:**
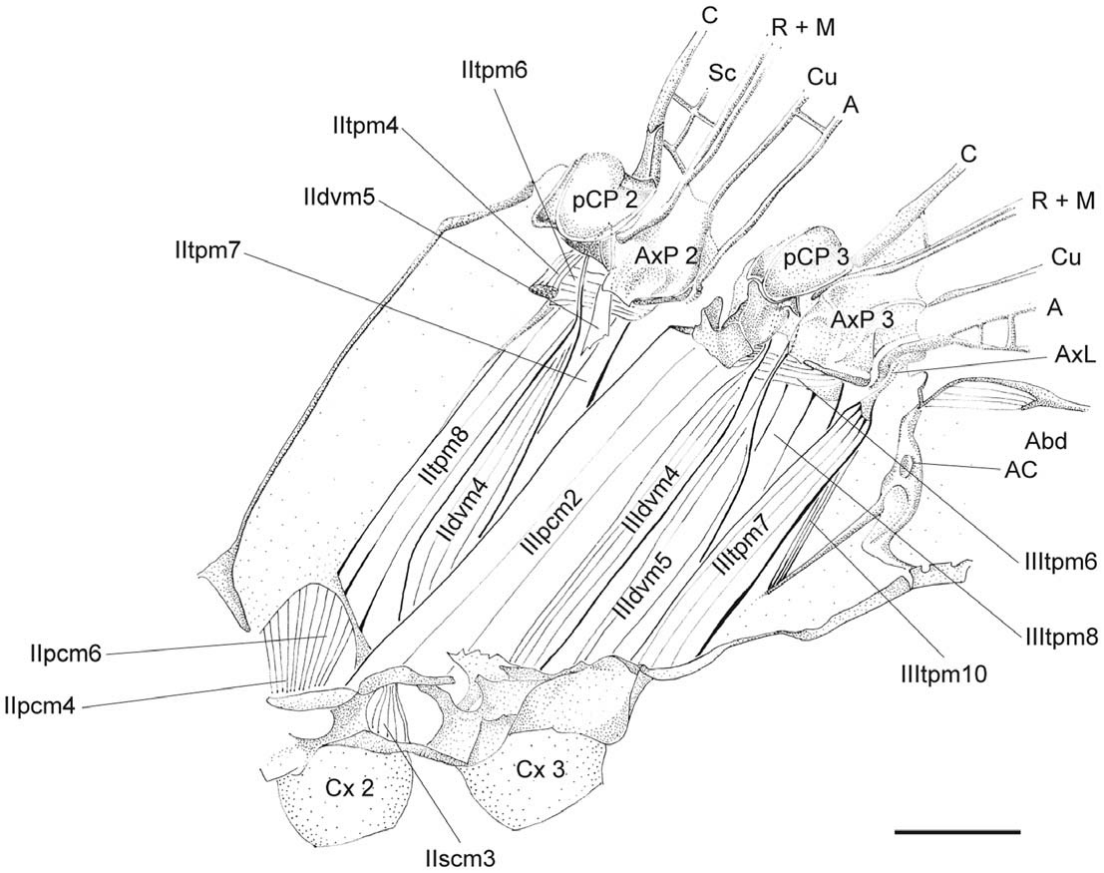
*C. splendens*, thorax musculature, longitudinal cut, right. Abbreviations: A – anal vein, Abd – abdominal segment, AC – antecosta, AxL – axillary ligament, AxP – axillary plate, C – costa, Cu – cubitus, Cx – coxa, M – media, pCP – proximale costal plate, R – radius, Sc – subcosta.

**Figure 12:**
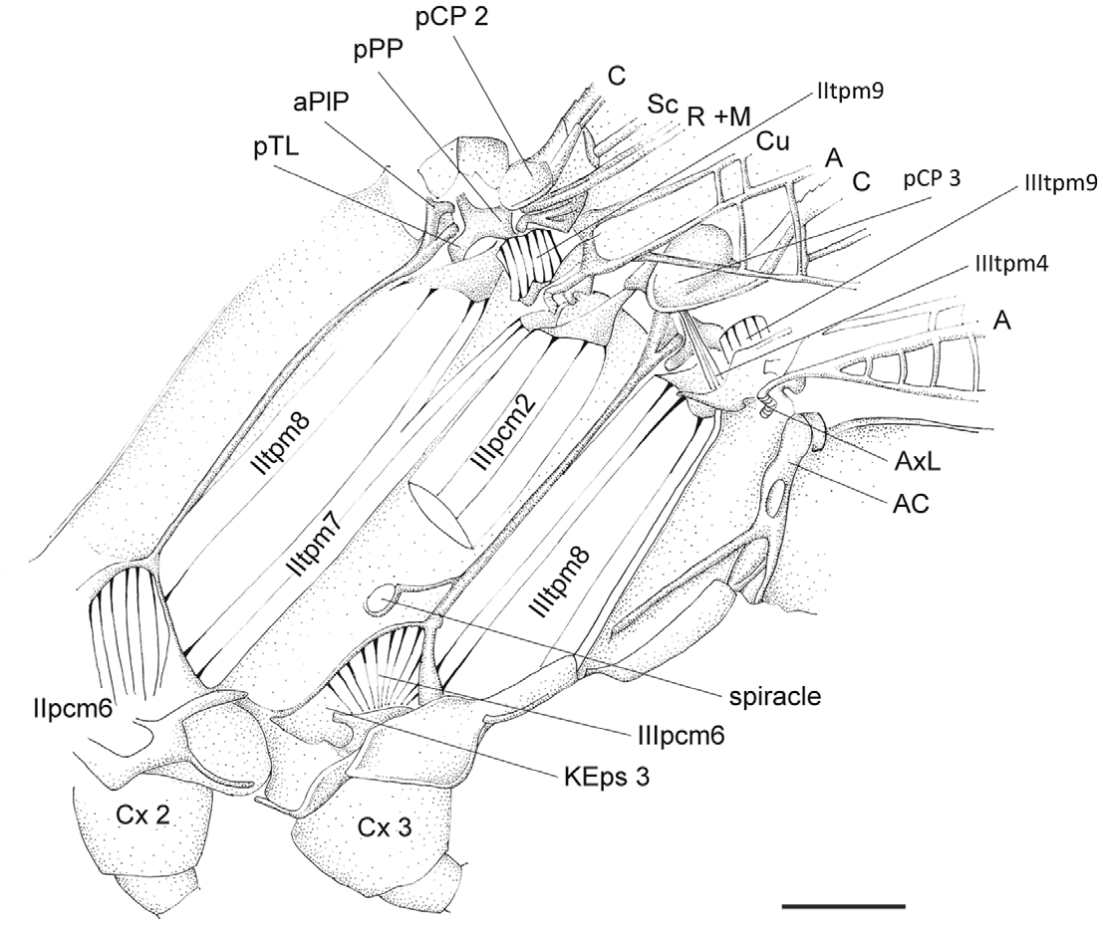
*C.splendens*, lateral thorax musculature, longitudinal cut, right. Abbreviations: A – anal vein, AC – antecosta, aPlP - anterior pleural process, AxL – axillary ligament, C – costa, Cu – cubitus, Cx – coxa, KEps – katepisternum, M – media, pCP – proximale costal plate, pPP – posterior pleural process, pTL - posterior tergal levler, R – radius, Sc – subcosta.

Together with the description of the muscles found, we already present our homology hypothesis by using the muscle names as proposed for Neoptera by Friedrich & Beutel [18]. We are aware that this presents a mixture of description and interpretation. However, stricter separation of these aspects would not support a clear and easily understandable presentation of the results.

For establishing our homology hypotheses we supplemented our data with information from the literature [7], [10], [14], [17], focusing on Asahina’s comprehencive study of *Epiophlebia superstes* [7], which represents a conspicuous mixture of anisopteran and zygopteran characters [4], [7], [20], [21]. Furthermore, in many aspects *Epiophlebia* seems to have the most ancestral character distribution within the Odonata (e.g. [22]).

For the skeletal elements of the thorax the nomenclature by Asahina [7] is used. Where necessary, this is supplemented by Snodgrass [6] and Ninomiya and Yoshizawa [14].

The homologies as well as the presence or absence of each muscle are listed in Table 1. In the muscle descriptions Asahina’s muscle numbers are given in square brackets after the name of each muscle. For mesothoracic muscles Asahina’s numbers for the corresponding metathoracic muscles are added in parentheses. The muscles are listed due to their occurrence in the pterothorax, from anterior to posterior. An additional table comparing our results with data from several other publications is available as supporting information (Table S1).

**Table 1:** Muscle homologies.

Homologies (Friedrich & Beutel (2008) and this study)		Asahina (1954)	
Name	Abbr.	Name	No. (Metath.)
**Mesothorax**			
M. mesanepisterno-trochantinalis	IIpcm1	Sternopleural (Sternobasalar)	21 (43)
M. mesobasalare-trochantinalis	IIpcm2	Sternopleural (Sternobasalar)	22 (44)
M. mesonoto-trochantinalis posterior	IIdvm3	Tergosternal (anterior tergosternal)	23 (46)
M. mesonoto-sternalis	IIdvm1	Tergosternal (anterior tergosternal)	23' (46')
M. prophragma-mesophragmalis	IIdlm1	Dorsal (lateral dorsal)	25 (45)
M. mesonoto-coxalis anterior	IIdvm4	Coxal (Coxobasalar)	26 (48)
M. mesonoto-coxalis posterior	IIdvm5	Coxal (Coxobasalar)	27 (49)
M. mesonoto-pleuralis anterior	IItpm4	Tergopleural	28 (50)
M. mesopleura-praealaris	IItpm2	–	–
M. mesepimero-axillaris tertius	IItpm9	Tergopleural (pleuro- RAP)	29/30 (51/52)
M. mesonoto-pleuralis posterior	IItpm6	Tergopleural (pleuro-RAP)	31 (53)
M. mesepimero-axillaris secundus	IItpm8	Tergopleural (pleurosubalar)	32 (54)
M. mesanepisterno-axillaris	IItpm7	Tergopleural (pleurosubalar)	33 (55)
M. mesepimero-subalaris	IItpm10	Tergopleural (pleurosubalar)	34 (56)
M. mesanepisterno-coxalis posterior	IIpcm4	Coxal (pleurocoxal)	36 (58)
M. mesofurca-coxalis medialis	IIscm3	Coxal (sternocoxal)	38 (61)
M. mesopleura-trochanteralis	IIpcm6	Trochanteral (Pleurotrochanteral)	39 (62)
M. mesofurca-trochanteralis	IIscm6	Trochanteral (Pleurotrochanteral)	40 (63)
M. profurca-mesofurcalis	Ivlm7	Ventral	41
**Metathorax**			
M. metanepisterno-trochantinalis	IIIpcm1	Sternopleural (Sternobasalar)	43
M. metabasalare-trochantinalis	IIIpcm2	Sternopleural (Sternobasalar)	44
M. mesophragma-metaphragmalis	IIIdlm1	Dorsal (lateral dorsal)	45
M. metanoto-phragmalis	IIIdlm2	Dorsal (lateral dorsal)	45'
M. metanoto-trochantinalis	IIIdvm3	Tergosternal (anterior tergosternal)	46
M. metanoto-sternalis	IIIdvm1	Tergosternal (anterior tergosternal)	46'
M. metanoto-coxalis anterior	IIIdvm4	Coxal (Coxobasalar)	48
M. metanoto-coxalis posterior	IIIdvm5	Coxal (Coxobasalar)	49
M. metanoto-pleuralis anterior	IIItpm4	Tergopleural	50
M. metapleura-praealaris	IIItpm2	–	–
M. metapimero-axillaris tertius	IIItpm9	Tergopleural (pleuro- RAP)	51/52
M. metanoto-pleuralis posterior	IIItpm6	Tergopleural (pleuro-RAP)	53
M. metepimero-axillaris secundus	IIItpm8	Tergopleural (pleurosubalar)	54
M. metanepisterno-axillaris	IIItpm7	Tergopleural (pleurosubalar)	55
M. metepimero-subalaris	IIItpm10	Tergopleural (pleurosubalar)	56
M. metanepisterno-coxalis posterior	IIIpcm4	Coxal (pleurocoxal)	58
M. metafurca-coxalis medialis	IIIscm3	Coxal (sternocoxal)	61
M. metapleura-trochanteralis	IIIpcm6	Trochanteral (Pleurotrochanteral)	62
M. metafurca-trochanteralis	IIIscm6	Trochanteral (Pleurotrochanteral)	63
Tendon	–	Ventral (Profurcoabdominal) Tendon	64
M. metaspina-abdominosternalis	IIIvlm3	Ventral (Profurcoabdominal)	66
M. metafurca-phragmalis	IIIdvm8	Tergosternal (posterior tergosternal)	67
M. mesofurca-abdominosternalis	IIIvlm2	Ventral	68

Since the prothorax has no active role in flight, it is omitted in this study.

### Musculature of the Pterothorax

In the following we describe 44 muscles, 19 muscles of the mesothorax and 23 muscles of the metathorax. Two previously undescribed muscles, M. mesopleura-scutalis proximalis (**IItpm2**) and M. metapleura-scutalis proximalis (**IIItpm2**), are described for *P. nymphula*, *C. puella*, *I. elegans*, *E. cyathigerum* and *P. latipes.* The presence of these two muscles in *P. pennipes* could not be confirmed.

### Musculature of the Mesothorax

**IIpcm1 -** M. mesanepisterno-trochantinalis [ = muscle no. 21 in Asahina’s nomenclature [7] (43 = corresponding muscle in metathorax)].

Origin: Preepisternum 2.

*P. nymphula* (Fig. 4), *C. splendens* (Fig. 10).

Insertion: Inserted with a long tendon at the anterior edge of proximale costal plate two (pCP2). The point of insertion is not exactly the edge but rather the membrane, which is connected with pCP2.

*P. nymphula* (Fig. 3, 4),

Characteristics: The muscle is short and thin and has a dorsal cap tendon. It is a direct tonic depressor muscle [10].

**IIpcm2 -** M. mesobasalare-trochantinalis [22 (44)].

Origin: Preepisternal apodem [7].

*P. nymphula* (Fig. 2), *C. splendens* (Fig. 9).

Insertion: Lateral to muscle IIpcm1 at the cranial edge of pCP2.

*P. nymphula* (Fig. 7E, 8B), *C. splendens* (Fig. 9).

Characteristics: It is a strong muscle with a dorsal cap tendon. In *Epiophlebia* [7] and in Anisoptera [17] this muscle was described as dichotomous, which is not the case in the species investigated herein. It is a direct flightmuscle [10].

**IIdvm1** - M. mesonoto-sternalis [23‘ (46‘)].

Origin: Distal, via a long tendon, at the preepisternal apodem to which IIdvm3 is attached.

*P. nymphula* (Fig. 2).

Insertion: At the tergum close to the tergal bridge, lateral of muscle IIdvm3.

*P. nymphula* (Fig. 2).

Characteristics: It is a short muscle distal from muscle IIdvm3. Its presence in *P. pennipes* could not be confirmed. The muscle is elongate in *P. latipes* compared to *P. nymphula.* In *C. splendens* it shows its maximal length.

**IIdvm3** - M. mesonoto-trochantinalis posterior [23 (46)].

Origin: Ventral of the apodem that originates at the inner wall of prefurca 2.

*P. nymphula* (Fig. 2), *C. splendens* (Fig. 9).

Insertion: Via a tendon, lateral at the mesoscutum between the tergal cone and tergal bridge [10] or at the inner bar of the tergal articulation [5].

*P. nymphula* (Fig. 2), *C. splendens* (Fig. 9).

Characteristics: The muscle has a dorsal cap tendon. It is an indirect tonic depressor of the wing [10].

**IIdvm4** - M. mesonoto-coxalis anterior [26 (48)].

Origin: Basal at the anterior part of the mesocoxa. In *C. splendens* more postero-lateral at the base of the mesocoxa.

*P. nymphula* (Fig. 1).

Insertion: Inserted with a tendon at the lateral part of the semi-detached scutal plate. In *C. splendens* at the upper edge of pCP2.

*P. nymphula* (Fig. 3), *C. splendens* (Fig. 10).

Characteristics: See muscle IIdvm5.

**IIdvm5** - M. mesonoto-coxalis posterior [27 (49)].

Origin: Basal at the anterior part of the mesocoxa, postero-median of muscle IIdvm4. In *C. splendens* caudal of muscle IIdvm4.

*P. nymphula* (Fig. 1).

Insertion: With a tendon at the proximal edge of axillary plate two (AxP2), or at the membrane between the mesoscutum and AxP2.

*P.nymphula* (Fig. 3), *C. splendens* (Fig. 10).

Characteristics: The muscles IIdvm4, IIdvm5 and IItpm9 are direct flight muscles. IIdvm4 and IIdvm5 are tonic lifters and IItpm9 is a phasic depressor [10]. IIdvm4 and IIdvm5 have cap tendons and are distinctly smaller than IItpm9. The insertion in *C. splendens* is located laterally and more ventral than in *P. latipes*. In all species studied the origin of muscles IIdvm4 and IIdvm5 is also the attachment point of the coxal musculature.

**IIdlm1** - M. prophragma-mesophragmalis [25 (45)].

Origin: Proximal end of the 3. tergal apophyse.

*P. nymphula* (Fig. 2), *C. splendens* (Fig. 10).

Insertion: Lateral at the posterior edge of the postnotum 2.

*P. nymphula* (Fig. 6A).

Characteristics: Broader than in *Epiophlebia* and in Anisoptera [7].

**IItpm4** - M. mesonoto-pleuralis anterior [28 (50)].

Origin: Pleural bar 2, close to the dorsal bifurcation.

*P. nymphula* (Fig. 1, 4).

Insertion: Median semi-detached scutal plate.

*P.nymphula* (Fig. 4), *C. splendens* (Fig. 11).

Characteristics: This muscle inserts at the lateral wall of the apodem of IIdvm3. It is an indirect tonic flight muscle [10].

**IItpm2** - M. mesopleura-praealaris (new muscle).

Origin: Dorsal region of pleuralbar 2, dorsal of muscle IItpm9.

*P. nymphula* (Fig. 4, 5, 7A).

Insertion: Median semi-detached scutal plate, posterior of IIpcm1.

*P.nymphula* (Fig. 4, 5).

Characteristics: This muscle is strongly developed in *E. cyathigerum* and in *I. elegans*. It is missing in *C. splendens* and L. viridis. Its presence in *P. pennipes* could not be confirmed. This muscle was not described for Odonata so far. It is thin and elongate and runs almost parallel to muscle IIpcm1. It assumes a similar function as IIpcm1 and/or is reinforcing it.

**IItpm9** - M. mesepimero-axillaris tertius [29/30 (51/52)].

Origin: With a short cap tendon at the posterior pleural process.

*P. nymphula* (Fig. 6B).

Insertion: In longitudinal axis at the ventral part of AxP2, precisely at the internal, caudal side of AxP2 next to the base of the anal vein.

*P.nymphula* (Fig. 6B, C).

Characteristics: IItpm4 and IItpm9 are located close together at AxP2, between the epifulcrum and the dorsal sclerite. Muscle IItpm4 is stronger and located more ventral; both have a cranial cap tendon. In *C. splendens* these muscles are distinctly separated from each other.

**IItpm6** - M. mesonoto-pleuralis posterior [31 (53)].

Origin: Lateral on the mesoscutellum, close to the proximal edge of AxP2.

*P.nymphula* (Fig. 5, 8A).

Insertion: Pleural bar between mesepisternum and mesepimeron, close to the dorsal bifurcation.

*P. nymphula* (Fig. 4, 5, 8A), *C. splendens* (Fig. 11).

Characteristics: In Anisoptera this muscle is attached to the lateral wall of the apodem where also muscle IIdvm3 inserts [10].

**IItpm8** - M. mesepimero-axillaris secundus [32 (54)].

Origin: Pleural bar between mesepimeron and katepisternum 2.

*P. nymphula* (Fig. 1, 4, 7A), *C. splendens* (Fig. 11).

Insertion: With a short tendon at the epifulcrum of AxP2.

*P.nymphula* (Fig. 4, 6D, 8A).

Characteristics: It is a broad and strong muscle with a dorsal cap tendon. This muscle is a direct depressor [10].

**IItpm7** - M. mesanepisterno-axillaris [33 (55)].

Origin: Ventral of muscle IItpm9.

*P. nymphula* (Fig. 1, 4, 6C), *C. splendens* (Fig. 11).

Insertion: With a short tendon at the postregion of AxP2.

*P.nymphula* (Fig. 4).

Characteristics: This muscle has a cap tendon and runs similar to IItpm9, but in comparison it is distinctly smaller. It is a direct depressor muscle [10].

**IItpm10** - M. mesepimero-subalaris [34 (56)].

Origin: In the middle at the pleural segmental border between meso- and metathorax.

*P. nymphula* (Fig. 1, 5, 6D, 8C).

Insertion: With a short tendon in the posterior region of AxP2, caudal of muscle IItpm6.

*P. nymphula* (Fig. 1, 5).

Characteristics: A short and thin muscle, with a dorsal, small cap tendon. The muscle is attached through resilin at the dorsal end [10].

**IIpcm4** - M. mesanepisterno-coxalis posterior [36 (58)].

Origin: Lateral side of the anterior edge of the mesocoxa.

*P. nymphula* (Fig. 1, 4), *C. splendens* (Fig. 11).

Insertion: Pleural bar between mesepisternum and katepisternum 2.

*P.nymphula* (Fig. 1, 4), *C. splendens* (Fig. 11).

Characteristics: A broad and flat muscle, running very close to katepisternum 2.

**IIscm3** - M. mesofurca-coxalis medialis [38 (61)].

Origin: Caudal at the basal side of the mesocoxa.

*P. nymphula* (Fig. 4).

Insertion: Ventral side of the furca branch 2.

*P.nymphula* (Fig. 7B).

**IIpcm6** - M. mesopleura-trochanteralis [39 (62)].

Origin: Similar to muscle IIpcm4, at the lateral side of the mesocoxa.

*P. nymphula* (Fig. 1, 4), *C. splendens* (Fig. 11).

Insertion: Similar to muscle IIpcm4, at the pleural bar between mesepisternum 2 and katepisternum 2.

*P. nymphula* (Fig. 4), *C. splendens* (Fig. 11).

Characteristics: IIpcm4 and IIscm3 run in parallel with IIpcm4 being slightly more laterally.

**IIscm6** - M. mesofurca-trochanteralis [40 (63)].

Origin: Latero-caudal at the base of the trochanter 2.

*P. nymphula* (Fig. 4), *C. splendens* (Fig. 11).

Insertion: At the proximal side of the prefurca 2.

*P. nymphula* (Fig. 7B), *C. splendens* (Fig. 11).

Characteristics: Lateral of muscle IIpcm4.

**Ivlm7** - M. profurca-mesofurcalis [41].

Origin: Furca-branch 2.

*P. nymphula* (Fig. 2, 7B).

Insertion: Furca 1.

*P. nymphula* (Fig. 2, 7B).

**42** Ventral (profurco-abdominal) M.

This structure has been described as a muscles [7], however, in the adult Zygoptera at this position only a tendon-like structure could be identified (cf. Asahina’s 64 in metathorax).

### Musculature of the Metathorax

**IIIpcm1** - M. metanepisterno-trochantinalis [43].

Origin: With a long tendon at the segmental border between epimeron 2 and episternum 3.

*P. nymphula* (Fig. 1, 4).

Insertion: With a long tendon at the membrane of proximal coxal plate three (pCP3).

*P. nymphula* (Fig. 1, 4).

Characteristics: A short muscles with cap tendons at both ends. These cap tendons are each attached to the cuticle through long tendons.

**IIIpcm2** - M. metabasalare-trochantinalis [44].

Origin: Preepisternal apodem 3 [7].

*P. nymphula* (Fig. 2), *C. splendens* (Fig. 10, 12).

Insertion: At the edge of the pCP3.

*P. nymphula* (Fig. 3), *C. splendens* (Fig. 10).

Characteristics: The muscle has a dorsal cap tendon.

**IIIdlm1** - M. mesophragma-metaphragmalis [45].

Origin: Proximal end of the tergal apophysis 4.

*P. nymphula* (Fig. 2), *C. splendens* (Fig. 10).

Insertion: Dorsal of the antecosta between abdomen and thorax.

*P. nymphula* (Fig. 2), *C. splendens* (Fig. 10).

Characteristics: In *P. latipes* the muscle has a flattend end, it is broader in Zygoptera than in Anisoptera [10].

**IIIdlm2** - M. metanoto-phragmalis [45‘].

Origin: Scutellum, close to the base of the tergal apophysis 4.

*P. nymphula* (Fig. 2, 8E),

Insertion: Proximal end of the tergal apophysis 4, dorso-lateral of muscle IIIdlm1.

*P.nymphula* (Fig. 8E), *C. splendens* (Fig. 9).

Characteristics: This muscle is only present in the metathorax of Zygoptera and *Epiophlebia*. In *Epiophlebia* it is distinctly thinner [7].

**IIIdvm3** - M. metanoto-trochantinalis [46].

Origin: Broad at the postero-median region of the metascutum.

*P.nymphula* (Fig. 6E), *C. splendens* (Fig. 9).

Insertion: Cranial at the base of the coxa 3.

*P. nymphula* (Fig. 2), *C. splendens* (Fig. 9).

Characteristics: Very strong muscle.

**IIIdvm1** - M. metanoto-sternalis [46‘].

Origin: Lateral region of the metascutum, postero-median to muscle IIIdvm3.

*P. nymphula* (Fig. 3), *C. splendens* (Fig. 10).

Insertion: With a long tendon at the prefurca 3.

*P. nymphula* (Fig. 3), *C. splendens* (Fig. 10).

Characteristics: This muscle corresponds to muscle IIdvm1 of the mesothorax, but is distinctly stronger. The presence in *P. pennipes* could not be confirmed. In *C. spelndens* the muscle is thin and elongate, whereas, in *E. elegans* it is quite small. It is missing in *Epiophlebia* [7].

**IIIdvm4** - M. metanoto-coxalis anterior [48].

Origin: With a tendon caudal of the semi-detached scutal plate, below the anterior area of pCP3.

*C. splendens* (Fig. 11).

Insertion: Mesocoxal disk at the basal edge of the coxa 3.

*P. nymphula* (Fig. 7B, C), *C. splendens* (Fig. 11).

Characteristics: See muscle IIIdvm5.

**IIIdvm5** - M. metanoto-coxalis posterior [49].

Origin: With a tendon at axillary plate three (AxP3), proximo-caudal from muscle IIIdvm4 at the origin of the radius-media vein.

*C. splendens* (Fig. 11).

Insertion: Mesocoxal disk at the basal edge of the coxa 3, posterior to muscle IIIdvm4.

*P. nymphula* (Fig. 7B,C), *C. splendens* (Fig. 11).

Characteristics: IIIdvm4 and IIIdvm5 share the same point of origin at the coxa 3. Each muscle inserts via a long tendon. They have dorsal cap tendons and serve as direct flight muscles.

**IIItpm4** - M. metanoto-pleuralis anterior [50].

Origin: Pleural bar 3, close to the dorsal bifurcation.

*P. nymphula* (Fig. 4, 5), *C. splendens* (Fig. 9, 10).

Insertion: Median semi-detached scutal plate.

*P.nymphula* (Fig. 5).

Characteristics: This muscle inserts at the lateral wall of the apodem where also muscle IIIdvm3 is attached. It is an indirect tonic flight muscle [10]. The muscle is stronger than its relative in the mesothorax.

**IIItpm2** - M. metapleura-praealaris (new muscle).

Origin: Dorsal region of the pleural bar between episternum 3 and epimeron 3, dorsal of muscle IIItpm9.

*P. nymphula* (Fig. 5).

Insertion: Median semi-detached scutal plate.

Characteristics: This muscle is strongly developed in *E. cyathigerum* and in *I. elegans* but it is missing in *C. splendens* and *L. viridis*. Its presence in *P. pennipes* could not be confirmed. It is thin and elongate, runs almost parallel to IIpcm1 and was not described in Odonata so far. It assumes a similar function as muscle IIpcm1 and/or is reinforcing it.

**IIItpm9** - M. metapimero-axillaris tertius [51/52].

Origin: A short cap tendon at the posterior pleural process.

*P. nymphula* (Fig. 6D), *C. splendens* (Fig. 9, 10).

Insertion: In longitudinal axis at the ventral part of AxP 3, precisely at the internal, caudal side of AxP3 next to the base of the anal vein.

*P. nymphula* (Fig. 6D), *C. splendens* (Fig. 9).

Characteristics: Both muscles are located close together at AxP 3, between the epifulcrum and the dorsal sclerite. Muscle IIItpm4 is stronger and located more ventral; both have a cranial cap tendon. In *C. splendens* these muscles are distinctly separated from each other.

**IIItpm6** - M. metanoto-pleuralis posterior [53].

Origin: Proximal edge of AxP 3.

*P. nymphula* (Fig. 4), *C. splendens* (Fig. 11).

Insertion: Dorsally on the pleural bar 3.

*P. nymphula* (Fig. 4, 5), *C. splendens* (Fig. 11).

Characteristics: The muscle is stronger than its relative in the mesothorax.

**IIItpm8** - M. metepimero-axillaris secundus [54].

Origin: Bar between epimeron 3 and katepisternum 3.

*P. nymphula* (Fig. 1, 4, 6A, D, 7B), *C. splendens* (Fig. 12).

Insertion: Through a tendon at the epifulcrum of the AxP 3, at the elongation of the cubitus.

*C. splendens* (Fig. 12).

Characteristics: It is a broad and flat muscle, with a dorsal cap tendon.

**IIItpm7** - M. metanepisterno-axillaris [55].

Origin: Bar between epimeron 3 and katepisternum 3.

*P. nymphula* (Fig. 1, 4, 7B), *C. splendens* (Fig. 11).

Insertion: With a short tendon at the posterior region of the AxP 3, posterior to muscle IIItpm9.

*P. nymphula* (Fig. 6D), *C. splendens* (Fig. 11).

Characteristics: The muscle has a dorsal cap tendon.

**IIItpm10** - M. metepimero-subalaris [56].

Origin: Bar between epimeron 3 and poststernum 3 [7].

*P.nymphula* (Fig. 1), *C. splendens* (Fig. 11).

Insertion: With a short tendon at the posterior region of the AxP 3.

*P. nymphula* (Fig. 5, 6B, D).

Characteristics: It is a short and thin muscle, which is attached through resilin [10] at the dorsal end (cf. IItpm8).

**IIIpcm4** - M. metanepisterno-coxalis posterior [58].

Origin: Bar between the katepisternum 3 and episternum 3.

*P. nymphula* (Fig. 7B).

Insertion: Lateral of the posterior edge of the coxa 3.

*P. nymphula* (Fig. 1, 7B).

**IIIscm3** - M. metafurca-coxalis medialis [61].

Origin: Furca 3.

*P. nymphula* (Fig. 7B).

Insertion: Antero-lateral edge of the coxa 3.

*P. nymphula* (Fig. 2, 4, 7B).

**IIIpcm6** - M. metapleura-trochanteralis [62].

Origin: Bar between katepisternum 3 and episternum 3, median of the muscle IIIpcm4.

*P. nymphula* (Fig. 1, 7B), *C. splendens* (Fig. 12).

Insertion: Antero-lateral of the coxa 3.

*P. nymphula* (Fig. 1, 7B), *C. splendens* (Fig. 12).

Characteristics: In *Epiophlebia* this muscle inserts at the base of the trochanter 3 [7].

**IIIscm6** - M. metafurca-trochanteralis [63].

Origin: Proximal at the furca branch 3.

*P. nymphula* (Fig. 1, 7B).

Insertion: Base of the trochanter 3.

*P. nymphula* (Fig. 1, 2, 4, 7B).

**64** Ventral (Profurcoabdominal) **Tendon** (cf. 42 in mesothorax).

Origin: Furca 1.

*C. splendens* (Fig. 11).

Insertion: Bar between epimeron 3 and 1. abdominal sternite, lateral of muscle IIIvlm3.

*P. nymphula* (Fig. 1, 4), *C. splendens* (Fig. 10).

Characteristics: This structure has been described as a muscle [7]. In the Zygoptera investigated it shows no muscle fibers.

**IIIvlm3** - M. metaspina-abdominosternalis [66].

Origin: Caudal of the poststernum 3.

*P. nymphula* (Fig. 2), *C. splendens* (Fig. 9).

Insertion: Bar between 1. and 2. abdominal sternite.

*P. nymphula* (Fig. 2, 8E), *C. splendens* (Fig. 9).

Characteristics: In *C. splendens* this muscle is distinctly flatened caudally.

**IIIdvm8** - M. metanoto-phragmalis [67].

Origin: With a long tendon at the anterior edge of the furca invagination [7].

*P. nymphula* (Fig. 3, 7D), *C. splendens* (Fig. 11), P. latipes (Fig. 8D).

Insertion: Posterior edge of the 1. abdominal tergite.

*P. nymphula* (Fig. 3), *C. splendens* (Fig. 10).

Characteristics: This muscle has a dorsal cap tendon that inserts directly at the antecosta. The muscle is broader and shorter than in Anisoptera [10]. The cap tendon in *I. elegans* inserts ventral of muscle IIIscm6. In *L. viridis* the cap tendon is weakly developed.

**IIIvlm2** - M. mesofurca-abdominosternalis [68].

Origin: Proximal side of the prefurca (at the anterior part of the furca invagination [7] or at the intersegmental apophysis of the pleural sternite [23]).

*P. nymphula* (Fig. 2), *C. splendens* (Fig. 9).

Insertion: With a long tendon at the bar between epimeron 3 and 1. abdominal sternite.

*P.nymphula* (Fig. 2), *C. splendens* (Fig. 9).

Characteristics: This muscle has a distinctly elongated tendon. In *C. splendens* it is very thin but the sclerotisations of the cap tendon and of the tergal sclerite are stronger than in the other species investigated. The cap tendons in *L. viridis* are either sparsely developed (IIpcm1, IIpcm2, IIdvm3, IIItpm4, IIIdvm3) or abent (IIdvm4, IIdvm5, IIIdvm4, IIIdvm5).

## Discussion

Asahina [7] listed 51 thorax muscles for Odonata in general and 42 muscles for adult Zygoptera. Of these muscles 19 belong to the mesothorax with Zygoptera lacking muscles 35 and 37. The remaining 23 muscles belong to the metathorax where Zygoptera do not have muscle 47, 57 and 60. In our study the 42 muscle of Asahina [7] could be confirmed. Additionally, four muscles (IIpcm2, IItpm2, IIIpcm2, IIItpm2) were found that were previously not known for the Odonata (cf. [5], [7], [10], [23]). IIpcm2 and IIIpcm2 are present in all species studied, with the exception of *L. viridis* and *C. splendens*. In *P. pennipes* the condition is uncertain because of insufficient data.

The short and slender pleuro tergal muscles IItpm2 and IIItpm2 are run from the dorsal part of the pleural bar to the median semi-detached scutal plate (Fig. 7A). They have positions and directions similar to IItpm4 and IIItpm4. Therfore, we assume a similar or reinforcing function (cf. [10]).

A couple of observed origin and insertion points differ from Asahina’s [7] descriptions. For example, IIdlm1 inserts at the anterior edge of the postnotum 2, not at the lateral side of the scutum 3 [7]. The muscles IIdlm1, IIIdlm1 and IIIdlm2 have been identified as indirect flight muscles [24]. They originate at the tergal apophysis and were previously homologized with dorsal longitudinal muscles of the neopteran pterothorax [10]. In the ground pattern of the Neoptera the longitudinal muscles run between the phragmata [3]. The point of insertion of muscle IIdlm1 at the caudal edge of the postnotum, i.e. at the caudal end of the second thorax segment, is equivalent to the position of the phragma in Neoptera, which supports the homologization proposed.

In *C. splendens* IIdvm4 and IIdvm5 originate at the distal base of the mesocoxa (cf. [7]). In the other seven species investigated, these muscles originate rather cranial at the anterior part of the mesocoxa. Since *Mnais strigata*, which was studied by Asahina [7] and *C. spledens* both belong to Calopterygidae, the translocation of the point of origin may well be an apomorphy of this group.

The points of origin of the corresponding metathorax muscles IIIdvm4 and IIIdvm5 differ from previous descriptions [7] in all species investigated. They are located caudal not distal of the base of the metacoxa.

Further more, IIdvm4 has been described as attaching to the inner caudal angle of the costal plate 2. In the Zygoptera investigated, IIdvm4, like its metathoracic homolog IIIdvm4, is attached to the lateral side of the semi-detached scutal plate. The muscles do not attach at the wing articulation, rather at a tergal sclerite. Therefore, they have to be characterized as indirect not as direct flight muscles [10]. This also applies to the strong indirect lifter IIdvm3 (and IIIdvm3), which is a main flight muscle and is also attached to the tergum.

Similarly, the pleuro-tergal muscles IItpm4, IIItpm4, IItpm2, IIItpm2, IItpm9, IIItpm9, IItpm6, IIItpm6 are all indirect flight muscles in the morphological sense, because they all insert on pleural or tergal sclerites.

The remaining muscles (IIpcm1, IIIpcm1, IIpcm2, IIIpcm2, IIdvm5, IIIdvm5, IItpm8, IIItpm8, IItpm7, IIItpm7, IItpm10, IIItpm10) are direct flight muscles since they are directly connected via tendons to the costal plate or to the axillary plate.

Consequently, the flight musculature of the Zygoptera consists of direct and historically indirect flight muscles. However, as far as the functions of the dorso-ventrally arranged flight muscles are concerned, all are now acting as direct muscles.

The conspicuously long tendons (e.g. IIpcm1, IIIpcm1) are characteristic for the Zygoptera.

### Homology of the Musculature of the Pterothorax in Zygoptera and Neoptera

Already in the descriptive part of this work we used the muscle nomenclature suggested by Friedrich and Beutel [18] for a generalized neopteran thorax. In the following the homologization of the flight musculature of Zygoptera with that of Neoptera is explained further (cf. Table 1, S1).

#### Dorsolongitudinal musculature (dlm)

The tergal apophyses are intersegmental invaginations and therefore not homologous to the primary diaphragms of Neoptera [24], but presumably to the pseudo phragmata of other insects [25]. The zygopteran muscles IIdlm1, IIIdlm1 and IIIdlm2 originate at the tergal apophysis and their homology with the dorsolongitudinal musculature of Neoptera appears to be unequivocal [17].

#### Dorsoventral musculature (dvm)

The points of origin and insertion of the zygopteran dorsoventral muscles are usually shifted to some degree in comparision to Neoptera. The reasons for this are not so much functional modifications, but drastic changes in shape and size of the notum of Odonata in coparision to that of other Pterygota. Nevertheless, the functions of these muscles as elevators of the wings are preserved. Their positions in the thorax together with the relationships to other muscles allow for a well-supported homologization. The muscles IIdvm1 and IIdvm3, IIdvm4, IIdvm5 could be identified in the odonatan thorax.

#### Ventral musculature (vlm)

The ventral muscle system in the Zygoptera appears to be highly simplified. We could identify one unequivocal ventral longitudinal muscle only: Ivlm7 is identical in its origin and insertion to its neopteran relative [18]. It seems not to be present in the Anisoptera but was also found in the Ephemeroptera [17].

#### Tergopleural musculature (tpm)

The muscles IItpm6 and IItpm2 originate dorsally at the pleural bar. Muscle IItpm6 inserts below the proximal region of the axillary plate. In Neoptera IItpm6 inserts on the 3. axillary. The proximal area of the odonatan axillary plate has been homologized with the 3. axillary of Neoptera [14], which supports our identification of this muscle.

IItpm2 inserts on the median semi-detached scutal plate. Therefore, a homology with either the neopteran IItpm2 or IItpm4 seems to be possible.

An identification of this muscle as IItpm4 could be excluded, because in Neoptera IItpm4 inserts on the 1. axillary [18], which in Odonata corresponds to the anterior-proximal area of the axillary plate [14]. Since IItpm2 inserts on the subtegula or on the prealare sclerite in Neoptera, which correspond to the odonatan scutal plate, our homologization appears to be most probable.

The points of origin of IItpm9 and IIItpm9 at the pleural processes of their segments as well as the points of insertion on the axillary plates (homologous region see above) correspond well to the situation in the Neoptera and also in the Ephemeroptera [17].

Due to the virtually identical points of origin and insertion in the Neoptera [18] as well as in the Odonata the homologization of the metathoracic muscles IIItpm4 and IIItpm6 appears to be unequivocal.

#### Pleuro-coxal musculature (pcm)

The zygopteran muscles IIpcm1 and IIIpcm1 originate at the preepisternum of the corresponding segments at the anterior edge of the pCP. Due to the ventro-dorsal expansion of the pleura in Odonata, this sclerite is directed nearly ventrally. Therefore, the orientations of the muscles in the thorax differ from there relatives in the Neoptera. However, the points of origin and insertion together with the relation to other muscles support the homologization.

The zygopteran muscles IIpcm4, IIIpcm4, IIpcm6 and IIIpcm6 show the same points of origin and insertion as their neopteran counterparts. Together with functional considerations this supports the suggested homologization. Nevertheless, there is some variation in the points of insertion of IIpcm6 and IIIpcm6. In Zygoptera they insert on the trochanter of the corresponding segments, very close the insertion of IIpcm4 or IIIpcm4, respectively. In Anisoptera and in *Epiophlebia* these insertions are shifted to some degree [7].

In summary, our comparative investigation of the flight musculature of the Odonata shows that homologization with the flight musculature of Neoptera in most cases is realtively straightforward. Due to the significant modifications of the skeleton of the odonatan pterothorax many points of origin shifted in variing degrees. However, the general positions and orientations of the muscles are still persistent. It also became clear that the flight musculature of Zygoptera and of Odonata in general is composed of direct as well as indirect muscles as it is the case in the Neoptera. Those muscles that historically are indirect flight muscles work as direct flight muscles in the Odonata due to the modifications in their skelettal system, especially in the notal sclerites. With a well-supported homologization of the flight muscles between the Zygoptera (and consequently Odonata) and the Neoptera, this character system now can also be used to expand datasets for the analysis of phylogenetic relationships of all pterygote insects.

## Materials and Methods

Odonata: Zygoptera.

Coenagrionidae.

*Pyrrhosoma nymphula* (Sulzer, 1776): Billingshäuser Schlucht, Göttingen, Germany.
*Coenagrion puella* (Linnaeus, 1758): Billingshäuser Schlucht, Göttingen, Germany.
*Enallagma cyathigerum* (Charpentier, 1940): Billingshäuser Schlucht, Göttingen, Germany.
*Ischnura elegans* (Vander Linden, 1820): Billingshäuser Schlucht, Göttingen, Germany.

Calopterygidae.

*Calopteryx splendens* (Harris, 1782): Billingshäuser Schlucht, Göttingen, Germany and Villemur sur Tarn, France.

Platycnemidae.

*Platycnemis latipes* (Rambur, 1842): Barsac, France.
*Platycnemis pennipes* (Pallas, 1771): Barsac, France and Villemur sur Tarn, France.

Lestidae.

*Lestes viridis* (Vander Linden, 1825): Barsac, France.

All regulations concerning the protection of free-living species were followed.

All necessary permits were obtained for collecting Odonata at the Billingshäuser Schlucht, Göttingen, Germany (permission granted by “Untere Naturschutzbehörde” file reference AZ.67.2.5 Wei). For collecting Damselflies in France, no specific permits are required. The locations where the damselflies were collected are not privately owned or protected in any way. No endangered or especially protected species were collected.

The specimens were collected into 80% EtOH. Subsequently, they were fixed in Dubosq-Brasil fixative [26] and stored in 80% EtOH.

Specimens were studied, prepared and drawn with the help of a stereomicroscope (Zeiss Stemi SV11) with a camera lucida.

Synchrotron radiation micro computed tomography (SRµCT) was applied in order to generate data for three-dimensional reconstruction of the structures of interest. Prior to scanning, the samples were critical point dried (Balzer CPD030). The SRµCT data were generated at the Swiss Light Source (SLS) in Villigen (Switzerland), at the beamline TOMCAT, (Proposal no. 20080794 and Proposal no. 20100088, ThH) as well as at the Deutsches Elektronen Synchrotron (DESY) in Hamburg, Germany, (Proposal no. I-20090102, SB).

Three-dimensional reconstructions (processing and visualization) of the data were prepared with Amira^®^ 5.2. (Visage Imaging, Richmond, Australia). All images were subsequently processed with Photoshop CS3 (Adobe System Inc., San José, USA).

## Supporting Information

Table S1 **Homologisation of thoracic muscle nomenclatures used by several authors.** - absent/? uncertain.
(XLSX)
